# Microbial Community Structure of Activated Sludge in Treatment Plants with Different Wastewater Compositions

**DOI:** 10.3389/fmicb.2016.00090

**Published:** 2016-02-18

**Authors:** Nataliya M. Shchegolkova, George S. Krasnov, Anastasia A. Belova, Alexey A. Dmitriev, Sergey L. Kharitonov, Kseniya M. Klimina, Nataliya V. Melnikova, Anna V. Kudryavtseva

**Affiliations:** ^1^Water Problems Institute, Russian Academy of SciencesMoscow, Russia; ^2^Engelhardt Institute of Molecular Biology, Russian Academy of SciencesMoscow, Russia; ^3^Vavilov Institute of General Genetics, Russian Academy of SciencesMoscow, Russia

**Keywords:** activated sludge, bacterial communities, 16S rRNA, metabolic pathways, MetaCyc

## Abstract

Activated sludge (AS) plays a crucial role in the treatment of domestic and industrial wastewater. AS is a biocenosis of microorganisms capable of degrading various pollutants, including organic compounds, toxicants, and xenobiotics. We performed 16S rRNA gene sequencing of AS and incoming sewage in three wastewater treatment plants (WWTPs) responsible for processing sewage with different origins: municipal wastewater, slaughterhouse wastewater, and refinery sewage. In contrast to incoming wastewater, the taxonomic structure of AS biocenosis was found to become stable in time, and each WWTP demonstrated a unique taxonomic pattern. Most pathogenic microorganisms (*Streptococcus, Trichococcus*, etc.), which are abundantly represented in incoming sewage, were significantly decreased in AS of all WWTPs, except for the slaughterhouse wastewater. Additional load of bioreactors with influent rich in petroleum products and organic matter was associated with the increase of bacteria responsible for AS bulking and foaming. Here, we present a novel approach enabling the prediction of the metabolic potential of bacterial communities based on their taxonomic structures and MetaCyc database data. We developed a software application, XeDetect, to implement this approach. Using XeDetect, we found that the metabolic potential of the three bacterial communities clearly reflected the substrate composition. We revealed that the microorganisms responsible for AS bulking and foaming (most abundant in AS of slaughterhouse wastewater) played a leading role in the degradation of substrates such as fatty acids, amino acids, and other bioorganic compounds. Moreover, we discovered that the chemical, rather than the bacterial composition of the incoming wastewater was the main factor in AS structure formation. XeDetect (freely available: https://sourceforge.net/projects/xedetect) represents a novel powerful tool for the analysis of the metabolic capacity of bacterial communities. The tool will help to optimize bioreactor performance and avoid some most common technical problems.

## Introduction

Domestic and industrial wastewater detoxification is of vital importance for the protection of natural ecosystems and human health. Wastewater processing is usually performed in several steps, including mechanical and biological treatment. The latter is crucial for the neutralization of chemical pollutants including toxicants and xenobiotics. Activated sludge (AS) is composed of aerobic and anaerobic microorganisms such as bacteria, archaea, fungi, and protists. It is capable of degrading organic compounds, including petroleum products, toluene, and benzopyrene (Seviour and Nielsen, 2010).

**Figure 1:**
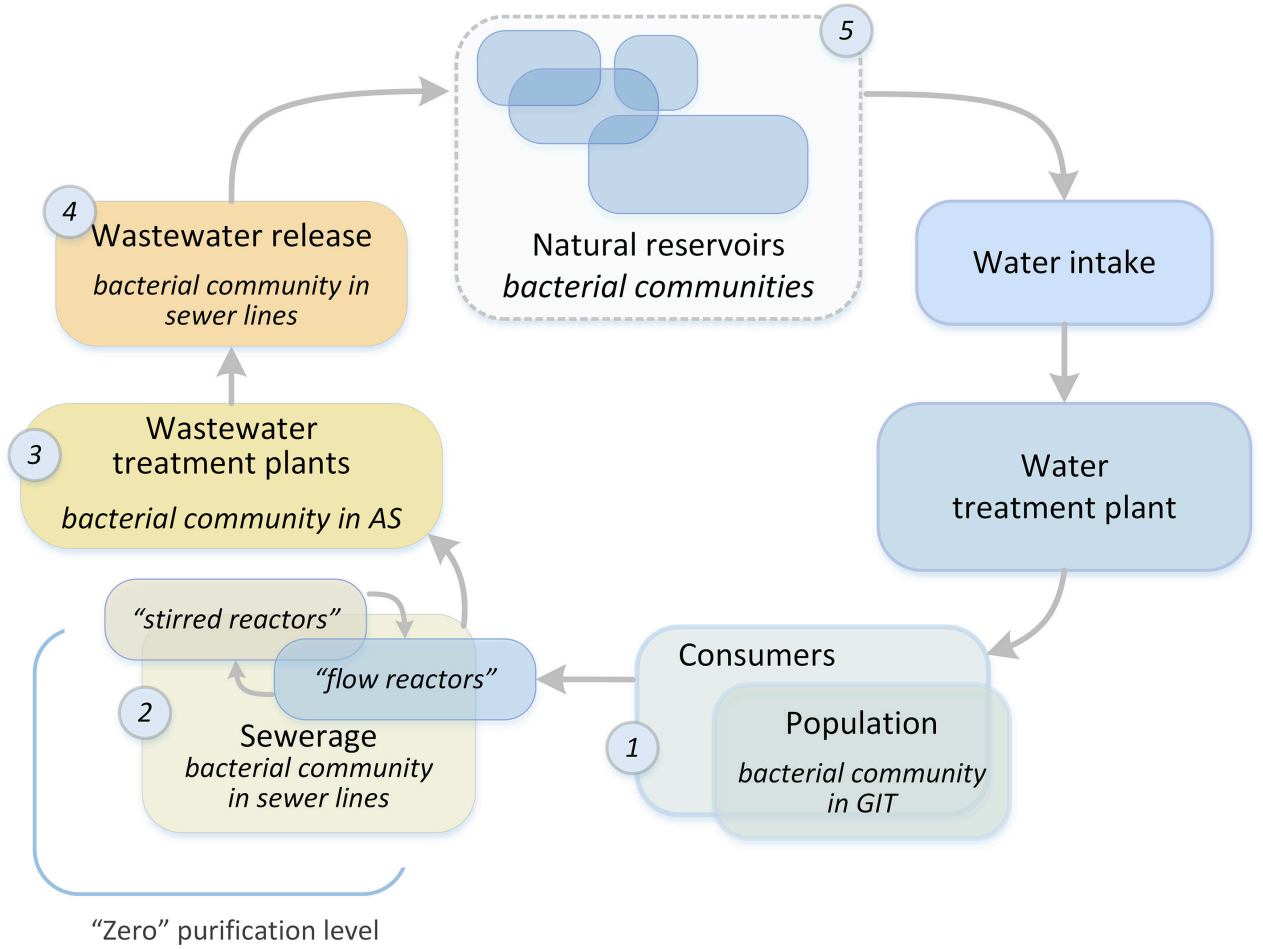
**The overall structure of the water system of a metropolis**. Places of bacterial community selection are marked with numbers: 1, human gastrointestinal tract; 2, sewerage system of a metropolis; 3, bioreactors of WWTPs; 4, treated wastewater discharge system; 5, natural reservoirs.

The process of water preparation for further treatment at plants begins in the sewerage system. The aquatic environment of a city is a system in which each subsystem is influenced by the subsystem upstream of it, and this opens up the possibility for preparing sewage for treatment at WWTPs. In order to optimize the purification process and enhance the elimination of xenobiotics and pathogenic microorganisms at the treatment plants, the sewer network should be considered as an initial wastewater treatment system (“zero” purification level, Figure 1). It is possible to create a technical framework for the formation of a given bacterial community (BC), which is already in the sewer network, by adding a special loading system to mixing chambers, and creating anaerobic or aerobic reactors (for example, at sewage pumping stations). This approach will significantly increase the time available for “useful” BCs to form in the wastewater. In most cases, WWTP and sewers have the same owner, thus facilitating the development and introduction of this innovative approach. To test the possibility of such an approach to water treatment, it is necessary to investigate the process of AS formation at WWTPs, namely, the dependence of the AS BC structure on the chemical and biological structure of incoming wastewater (IW). This is extremely important when it comes to the degradation of xenobiotics (for example, oil products) or the process of disinfection. The removal of these waste components is the most energy- and resource-consuming step, and there is still no reliable biological purification method to remove many xenobiotic pollutants.

The dynamically stable structure of most BCs depends on the incoming substrate-containing bacteria. Gastrointestinal tract (GIT) is one example of such systems; the gut BC is continuously supplied with nutrients and associated bacteria. However, the composition of the GIT BC tends to be stable over time, though it depends on the geographic place of residence and the nutrition (Neish, 2009; Gillevet et al., 2010; Yatsunenko et al., 2012; Eren et al., 2015). The stability of the GIT BC structure suggests that the incoming bacteria play a leading role only in the initial formation of the BC, whereas the final selection of predominant bacterial families is determined by the chemical composition of the nutrients (Arumugam et al., 2011; Nam et al., 2011; Wu et al., 2011; Yatsunenko et al., 2012). Further transformation of BC structures can be observed in wastewater transport systems. Despite the fact that stool is the main source of bacteria in sewage, most of the wastewater taxa were found to be consistently associated with the sewage infrastructure (Shanks et al., 2013; Newton et al., 2015), and only 12–15% of 16S rRNA gene sequences in the sewage are of human fecal origin (Shanks et al., 2013; Newton et al., 2015). However, 97% of fecal taxa are preserved in the sewage, and the relative content of the corresponding sequence tags mirrored the population structures of human fecal samples (McLellan et al., 2010; Newton et al., 2015). The next steps in BC transformation occur in the WWTP. Similarly, the impact of the wastewater chemical composition seems to be stronger than the influence of microorganisms introduced into the AS with the (IW; Ju and Zhang, 2015; Lee et al., 2015).

AS is a complex system with complex, interconnected trophic relationships between microorganisms. The biodiversity of AS is greater than that of IW (Lee et al., 2015), and uncultivated species are the most abundant (up to 60–90%) in various types of AS (Liaw et al., 2010; Tomazetto and Oliveira, 2013). This restricts direct microbiological studies of AS taxonomic structure, although 16S rRNA gene sequencing remains an option. Before the era of high-throughput sequencing methods, 16S rRNA genes PCR amplicon cloning and Sanger sequencing were required to perform 16S rRNA studies (Jeter et al., 2009). The introduction of novel high-throughput sequencing methods revolutionized the study of BC structure dynamics (Simon and Daniel, 2011).

WWTP BC structure is now becoming the focus of bioreactor operation monitoring. The monitoring of AS biological structures means many technical problems can be avoided. Filamentous bacteria are a necessary component of the AS, but their excessive growth results in AS bulking and foaming, two of the most common technical problems of WWTP bioreactor operation. Filamentous bacteria primarily consume carbohydrates and secrete many exo-enzymes, e.g., chitinase, glucuronidase, and galactosidase (Kragelund et al., 2007). 16S rRNA gene sequencing identified *Gordonia* species as the most abundant in the analyzed AS. The application of four bacteriophages, which target *Gordonia*, reduced *Gordonia* abundance 10-fold and eliminated these problems (Liu et al., 2015). Based on 16S rRNA gene sequencing, functional interactions between various groups of bacteria that perform nitrogen fixation, nitrification, ammonification, and other biochemical processes have been shown for an entire WWTP bioreactor, and the temporal dynamics of BCs have also been studied (Wells et al., 2014; Ju and Zhang, 2015).

The aim of the present study was to analyze AS microbial community structure across different WWTPs with IW of different chemical compositions: (1) municipal wastewater (MW), (2) MW and slaughterhouse wastewater, and (3) MW and refinery sewage enriched with petroleum products. We evaluated the dynamics of AS BC taxonomic structures and the effect of microorganisms introduced with the incoming sewage on the formation of AS microbial composition.

In the present work, we introduce a novel approach enabling the inference of metabolic pathways that are available in a BC. In contrast to other methods of BC metabolic profiling, this approach does not need complete-metagenome sequencing and is capable of inferring metabolic pathways based only on BC taxonomic data.

## Materials and methods

### Site description and sampling procedure

The samples were obtained from three wastewater treatment plants (WWTPs) in the Moscow region (Russia). Each WWTP treats 50,000–60,000 m^3^ of wastewater daily. The treated effluent is discharged into the tributaries of the Moskva-river basin. We selected three WWTPs with incoming wastewater of different chemical compositions. The first WWTP (WWTP-C, city) received only domestic wastewater. The second WWTP (WWTP-S, slaughterhouse) received effluent from slaughterhouses in addition to domestic wastewater, and the third WWTP (WWTP-P, petroleum) received domestic wastewater and refinery effluent with high petroleum-product content. These WWTPs used the same technical approach: mechanical treatment, a primary sedimentation tank, an aeration tank with complete biological treatment (without nutrient removal), and a secondary clarifier.

Sampling for BC structure studies was performed three times from October 2013 to December 2013 (1 month interval) in the chambers of incoming sewage and in the aerotanks with the AS. The samples were taken from the same locations in each WWTP. The similarity of the structures was estimated for samples that were collected in 1 day but with a time delay (15 min). Next, we compared the structure of the AS and the IW samples from the three WWTPs mentioned above. In accordance with the wastewater treatment scheme, aerotanks of all WWTPs are aerated all over their length. Samples for BC studies were taken in BD Falcon tubes from the central part of the aerotanks, and placed in liquid nitrogen within 20 min. Samples for chemical studies were taken from the same locations in the aerotanks and immediately transferred to the WWTP's laboratory.

### Study of the chemical composition of incoming waste

The study of the BC environment was based on the evaluation of the chemical composition and the physical parameters of the incoming sewage. Three sewage treatment plants were regularly analyzed and the following parameters were measured: temperature, suspended solids, solids, biochemical oxygen demand (BOD_5_), chemical oxygen demand (COD), total nitrogen, ammonia nitrogen, nitrite nitrogen, nitrate nitrogen, phosphates, petroleum products, synthetic surfactants, iron, sulfates, chlorides, fats, and phenol. The chemical analysis was performed using EPA Standard Methods for the Examination of Water and Wastewater (APHA; Clesceri et al., 1999). Petroleum products were determined by using an IR spectrophotometer. The sampling was performed once per month.

### DNA isolation, 16S rRNA gene amplification, sequencing

DNA isolation was performed using PowerSoil® DNA Isolation Kit (MO BIO Laboratories, CA USA) according to the manufacturer's protocol. The kit is intended for the analysis of environmental samples containing humic acids including complex types of soil, such as compost, sludge, and manure. PowerSoil allows efficient elimination of PCR inhibitors contained in the environmental samples. The environmental samples were placed into a bead beating tube for rapid and thorough homogenization. The DNA extracts were stored at –20°C. NanoDrop ND-1000 (Thermo Fisher Scientific, MA USA) was used to assess DNA concentration and detect possible contaminations (A260/A280 ratio). We used 16 rRNA PCR amplification primers fused with GS Junior sequencing adapters: 5′-CCA TCTCATCCCTGCGTGTCTCCGACTAG-barcode-GTGCCAGCMGCCGCG GTAA-3′ (forward, F515) and 5′-CCT ATCCCCTGTGCCTTGGCAGTCTCAGGGACTACVSGGGTATCT AAT-3′ (R806, reverse). The barcodes had a length of 10 nucleotides. These primers target the V4 hypervariable region (Bates et al., 2011; Caporaso et al., 2011). Each reaction was run with 25 μL mix using KAPA HiFi PCR kit (KAPA Biosystems, USA). PCR was conducted under the following conditions: 15 min at 95°C, then 25 cycles of amplification (10 s at 95°C, 15 s at 58°C, 15 s at 72°C), and then 5 min at 72°C. The amplicons were purified with MiniElute Gel extraction kit (Qiagen, Germany) and Agencourt AMPure Beads (Beckman Coulter, CA USA). Emulsion PCR was carried out with GS Junior Titanuim emPCR Kit (Lib-L, Roche, Switzerland) according to the manufacturer's instructions. Sequencing was performed using the GS Junior system (Roche) at the EIMB RAS “Genome” Center (Moscow, http://www.eimb.ru/rus/ckp/ccu_genome_ce.php).

### Bioinformatics

#### Taxonomic analysis

The obtained sequences were trimmed using Trimmomatic (Bolger et al., 2014). Reads shorter than 250 bp were discarded. We used UCHIME to detect and eliminate potentially chimeric reads (Edgar et al., 2011). About 3–6% of reads were annotated as chimeric. Next, we used the Ribosomal Database Project (RDP) Classifier tools to perform taxonomic annotation up to genera level with adjustment for the gene copy number (Cole et al., 2014). This method allows for the presence of multiple 16S rRNA genes in a bacterial genome, and thus avoids over-representation of such taxa. The RDP Classifier's confidence threshold was set as 90%. Sequences that did not pass the threshold at the current taxonomic level were marked as unclassified (but retained at the upper level). The data were normalized by the total read count.

To assess the similarity of taxonomic structures between different samples, we calculated the distance matrix. The distance was evaluated as [1 – *r*], where *r* is the Pearson correlation coefficient between a pair of vectors $\left(x_{1}^{0.5},x_{2}^{0.5},\ldots \right)$ and $\left(y_{1}^{0.5},y_{2}^{0.5},\ldots \right)$ and *x*_*i*_ is the normalized read count for taxon *i* in sample *x*. We applied a square root transformation in order to increase the impact of low-abundance taxa. Agglomerative hierarchical clustering of samples was performed using Ward's minimum variance method (R, hclust package). PCA analysis was performed with the same vectors using the FactoMineR R package.

To evaluate the statistical significance of the temporal stability differences between AS and incoming sewage, we proceeded as follows. First, we collected distance values for all possible pairs of samples taken from one location at different times (replicates taken within 15 min were merged). Thus, six pools were formed: three pools for AS, and three pools for the incoming sewage. Each pool comprised three distance values (Oct vs. Nov, Oct vs. Dec, and Nov vs. Dec). Then we integrated pools for AS and IW, and performed Mann-Whitney U tests for these two groups of values.

Heatmaps and dendrograms were generated using the ggplot2 R package. Wordcloud and taxonomic tree representations of BC structure were created using MEGAN5 (Huson and Weber, 2013).

#### Analysis of the metabolic capability of bacterial communities

BCs of AS are complex biosystems with intertwined trophic links. The degradation of almost all xenobiotics or other toxicants is a multi-step process involving many enzymes. In many cases, just one species of microorganism is not capable of performing all the steps required for complete biodegradation. The efficient elimination of pollutants is possible only in diverse communities. We developed XeDetect software to analyze the biodegradational potential of different BCs.

XeDetect considers a community of microorganisms as a biosystem in which microbial populations are capable of performing a specific set of biochemical reactions. XeDetect uses the MetaCyc database (Caspi et al., 2014) to reveal sets of enzyme-catalyzed reactions available for microorganisms, e.g., species and genera that were identified during 16S rRNA genes sequencing studies. Then, XeDetect identifies the metabolic pathways that include these reactions.

For example, we want to estimate the availability of a metabolic pathway, which contains *P* sequential enzyme-catalyzed reactions *R*_*i*_. It is assumed that the reaction *R*_*i*_ can be performed by *C*_*i*_ organisms. The organism *j* is represented with *N*_*i, j*_ 16S rRNA genes amplicon reads, and the relative rate of the reaction for organism *j* is *u*_*i, j*_. In this case, the overall pathway speed *v* is proportional to

$$\begin{array}{l} v\sim {\left(\sum_{i=1}^{P}{\left(\sum_{j=1}^{C_{i}}N_{i,j}u_{i,j}\right)}^{-1}\right)}^{-1} \end{array}$$

This formula does not regard the compartmentalization of chemical processes; reactions can occur in different organisms, and the speed of transport of intermediate metabolites can have a significant impact. Neither this factor nor individual reaction rates *u*_*i, j*_ are easily estimable. However, one can discount these factors if one only wishes to evaluate the availability of a metabolic pathway. In XeDetect, we introduce the *availability score* (*S*_*A*_), which is calculated as:

$$\begin{array}{l} S_{A}\sim {\left(\sum_{i=1}^{P}{\left(\sum_{j=1}^{C_{i}}N_{i,j}\right)}^{-1}\right)}^{-1} \end{array}$$

If some stages of a pathway represent a series of parallel subpathways (or reactions), XeDetect first calculates *S*_*A, sub*_ for each of these parallel subpathways, and then computes the overall *S*_*A*_ according to the above formula, with *N*_*i, j*_ = *S*_*A, sub*_.

Thus, in contrast to other tools (such as MEGAN), XeDetect infers metabolic potential based only on taxonomic structures derived from 16S rRNA genes sequencing. XeDetect does not require complete metagenome sequencing. XeDetect is freely available at https://sourceforge.net/projects/xedetect. A screenshot of the XeDetect user interface is provided in Figure S1. However, it should be noted that *S*_*A*_ scores are only estimates; they should not be regarded as exact values.

Using XeDetect, we predicted the metabolic potential of 15 AS samples (five samples per each WWTP). Availability scores (*S*_*A*_) were calculated for 2515 pathways available in MetaCyc. Among them, there were 729 pathways with “degradation” as a keyword. Next, using the *t*-test and ANOVA, we identified pathways characterized by different *S*_*A*_ across samples of three WWTP (differentially represented pathways).

## Results

### Incoming wastewater as a substrate for the formation of different BC structures

**Figure 2:**
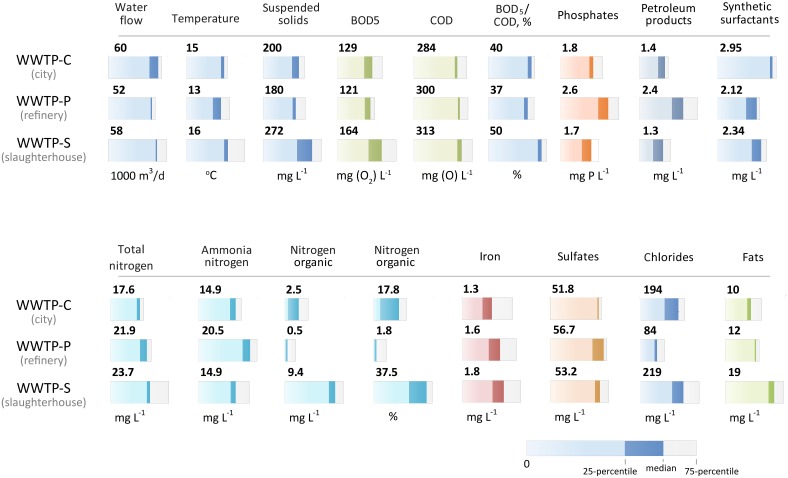
**Chemical composition of the incoming wastewater and activated sludge of three WWTPs**. The median value and quartile range are provided (explained under the figure). The chemical composition did not change significantly over the 2 years of observation.

The biochemical composition of the incoming sewage is an important factor in the selection of bacteria for populating aerotanks. The chemical ñomposition of the incoming wastewater for 2012–2013 (monthly sampling) is shown in Figure 2. The characteristics of the incoming sewages of all three WWTPs did not differ significantly in the following parameters: the temperature and the total concentrations of iron, sulfates, and detergents. We revealed key differences in the following parameters:

- WWTP-P incoming sewage had a 1.7-fold greater petroleum product content compared to two other WWTPs;
- WWTP-S incoming sewage had a 2.5–20-fold greater organic nitrogen content (in absolute and percentage terms);
- The highest ammonia nitrogen content (20.5 mg/L) was found in WWTP-P. The two other WWTPs contained an average concentration of 15 mg/L;
- Increased BOD_5_ and COD, as well as high BOD_5_/COD ratio (>1.25), a twofold increase in phosphorus content, and in total and organic nitrogen content was observed in WWTP-S sewage;
- Chloride content was different at all three WWTPs: the WWTP-P contained the lowest concentration (85 mg/L), and the two other WWTPs had about 200 mg/L.

These trends were noted throughout the observation (2 years). WWTP-C wastewater had the greatest chemical composition stability over time. The incoming sewage of WWTP-S was characterized by its physical structure heterogeneity (the abundance of suspended particles of various sizes).

### The structure of microbial communities

**Figure 3:**
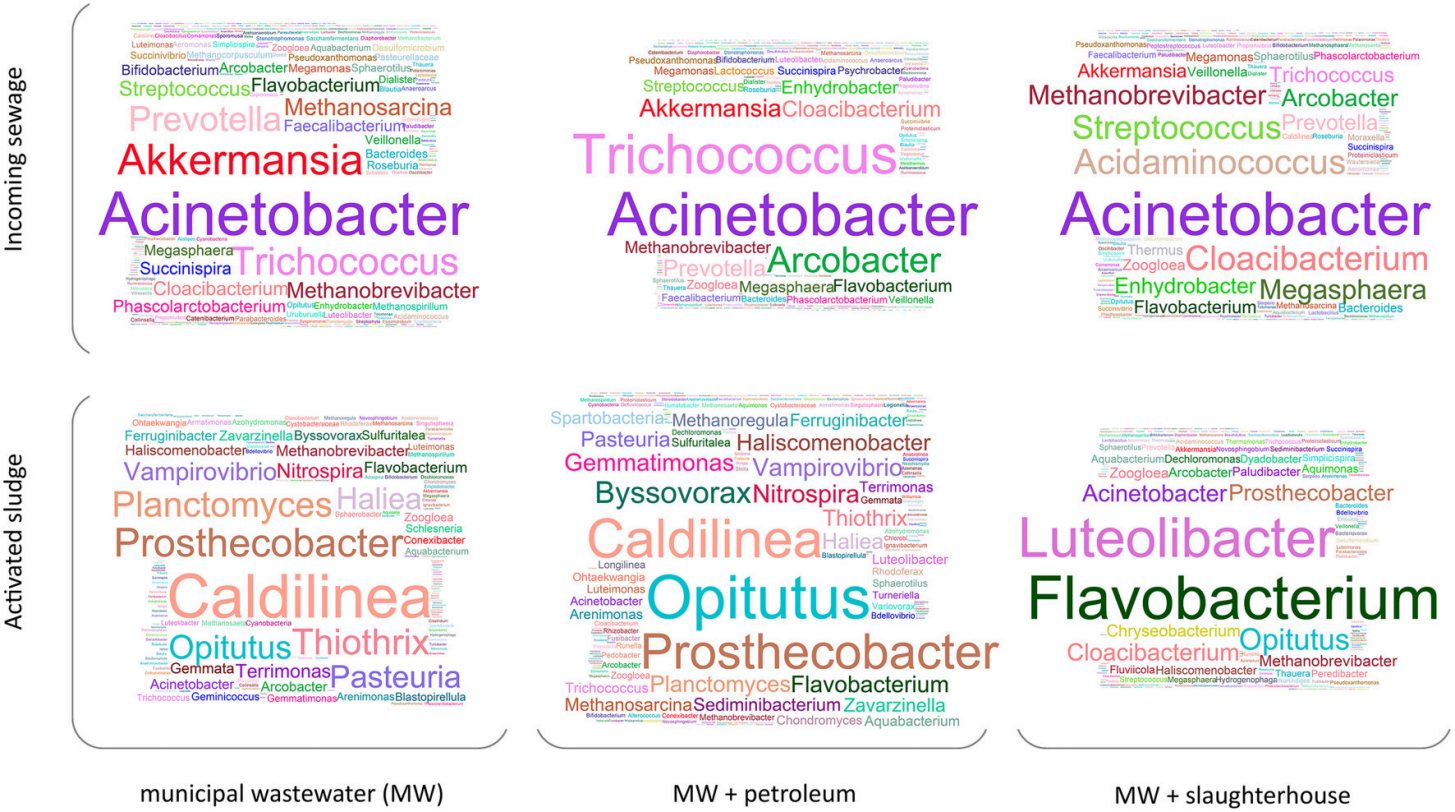
**The structure of bacterial communities of the incoming sewage and activated sludge for three WWTPs (averaged across replicates and then across monthly periods)**. The size of letters is proportional to the square root of taxon relative content (at genera level).

A total of 771 and 913 operational taxonomic units (OTUs) have been identified for AS and incoming wastewater samples, respectively (1063 overall OTUs). These OTUs represent 48 classes, 172 families, and 474 genera of microorganisms. Figure 3 illustrates the relative content (e.g., normalized with total 16S rRNA genes read count) of microorganisms at genera level. As can be seen from Figure 3, *Acinetobacter* is the most abundant genus in all three types of incoming sewage. There are many genera predominantly found in only one type of sewage: *Akkermansia* and *Prevotella* are mostly encountered in domestic wastewater; *Acidaminococcus, Cloacibacterim*, and *Megasphaera*, in slaughterhouse wastewater; and high levels of *Arcobacter* are observed in municipal wastewater with petroleum products.

**Figure 4:**
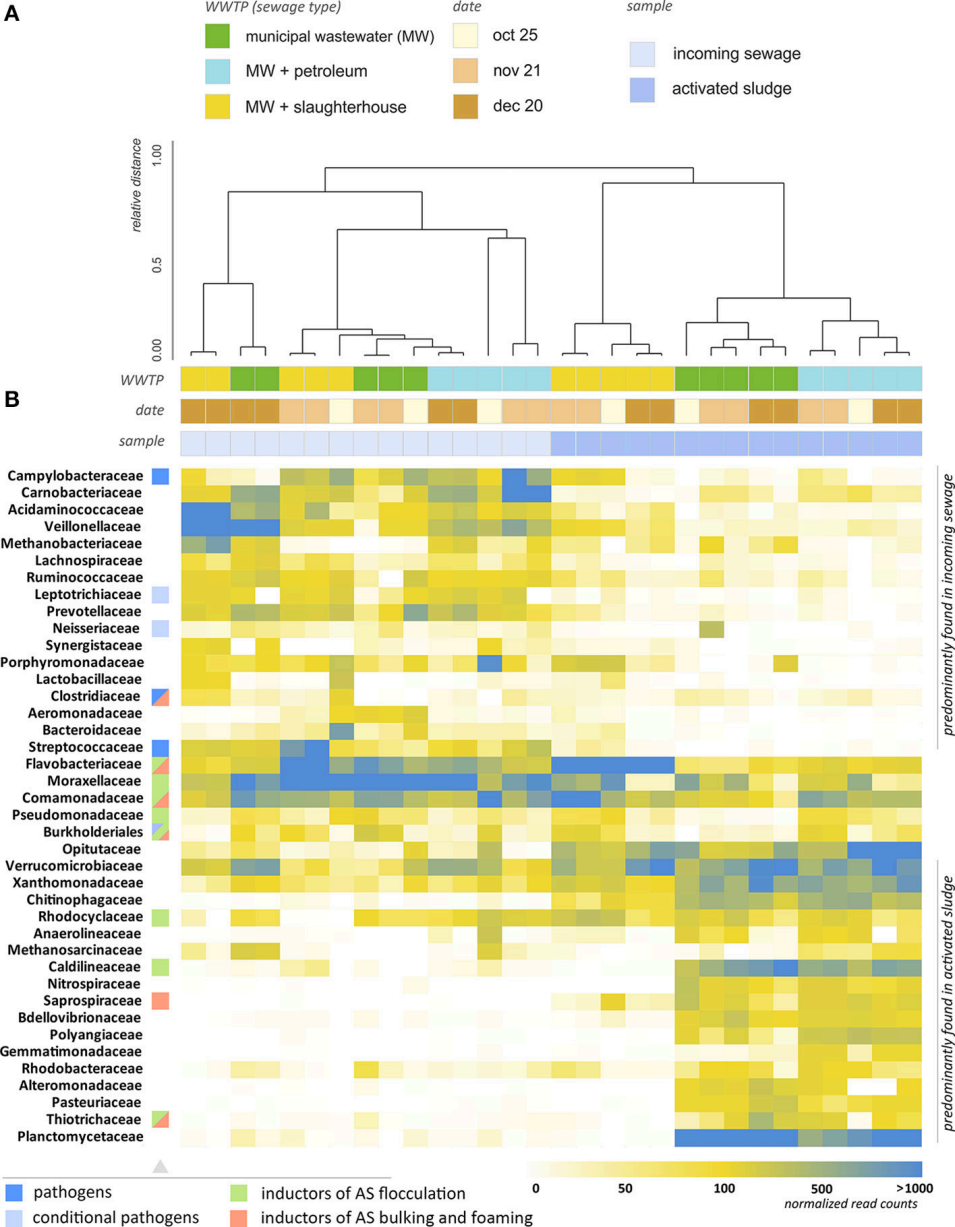
**Analysis of the taxonomic structure of activated sludge and incoming wastewater. (A)** Dendrogram illustrating the results of hierachial clustering of AS and IW samples (at the level of families). Two major clusters are identified—groups of AS and IW samples. Clustering with the lower distance thresholds reveals similarity of AS samples for each WWTP taken at different times. This indicates the temporal persistence of their bactericenosis, whereas the structure of IW is unstable over time. Microbial structures of WWTP-P and WWTP-C activated sludges are close, and WWTP-S AS is an outlier. **(B)** Heatmap showing the relative content of microorganisms in the samples (normalized by total 16S rRNA read count). The families containing pathogens, inductors of AS flocculation, bulking and foaming are marked with colored squares.

Figure 4B provides a heatmap representation of the relative content of microorganisms (at family level) across 30 samples of AS and IW, including replicates. The heatmap includes the top 40 bacterial families, which represent 94.2–97.5% of all bacterial 16S rRNA genes reads. As can be seen from Figure 4A, clustering of the samples at the family level splits them into two major groups: those in samples of IW, and those in AS. Cluster analysis also suggests a pronounced difference between BC of WWTP-S AS and AS from the other two WWTPs. WWTP processing MW with slaughterhouse wastewater (WWTP-S) demonstrated a strikingly different taxonomic profile compared to the other WWTPs. The average distance (*ad*) between WWTP-S and the other WWTP is 0.332. A significant proportion of the bacterial families that are highly abundant in WWTP-C and WWTP-P were found in a dramatically lower proportion in WWTP-S-AS. Activated sludge of WWTP-S demonstrated a 30–100-fold increase in the relative content of *Flavobacteriaceae*, and a 50–500-fold decrease in *Caldilineaceae, Nitrospiraceae, Planctomycetaceae*, and many other bacterial families, compared to WWTP-C and WWTP-P. Of the top 100 families, 22 demonstrated at least a fivefold decrease in their relative content in WWTP-S, and 32—at least a twofold decrease, compared to the populations in the incoming wastewater.

AS from the WWTP treating MW with petroleum products was characterized by a 3–10-fold increase in *Gemmatimonas* (polyphosphate-accumulating bacteria), *Methanosarcinales* (methane-consuming archaea), *Pseudomonadaceae, Polyangiaceae*, and *Comamonadaceae* compared to the WWTP treating MW only. Thus, each of these WWTPs can be characterized by a unique taxonomic signature—a set of the most abundant bacterial families.

To evaluate the short-term and long-term stability of AS and IW BCs, we assessed the average intragroup distances for the samples as a measure of their dissimilarity. The distances were calculated taking into account all taxonomic levels. A heatmap of the distance matrix is shown in Figure S2. The greatest similarity was revealed for the replicates taken at 15-min intervals (*ad* = 0.022 ± 0.019). The average distance between the samples taken from one location at 1 month intervals varied from 0.03 to 0.08 (AS samples) to 0.10–0.16 (IW samples, Figure S2). The difference between these AS and IW samples is statistically significant (*p* = 0.01). Thus, AS has a microbial structure, which is more stable over a long period compared to that of IW.

The long-term variability of IW taxonomic profiles (*ad* = 0.149 ± 0.055) is quite comparable to their variability between different WWTPs (*ad* = 0.143 ± 0.088). However, the same does not hold true for the AS: while the AS taxonomic profiles of WWTP-C and WWTP-P are close to each other (*ad* = 0.065 ± 0.025), the profile of WWTP-S is strikingly different (*ad* = 0.33 ± 0.04 between WWTP-S and the two other plants), as mentioned above. This tendency is also observed at a family level (Figure 4A). While AS samples taken at different times are clustered closely, with a distance not exceeding 20% of the maximum observed value (across all the samples), the cluster analysis failed to group IW samples by their origin. IW samples are characterized by transient bursts of various bacteria including *Carnobacteriaceae, Veillonellaceae*, and *Porphyromonadaceae*.

**Figure 5:**
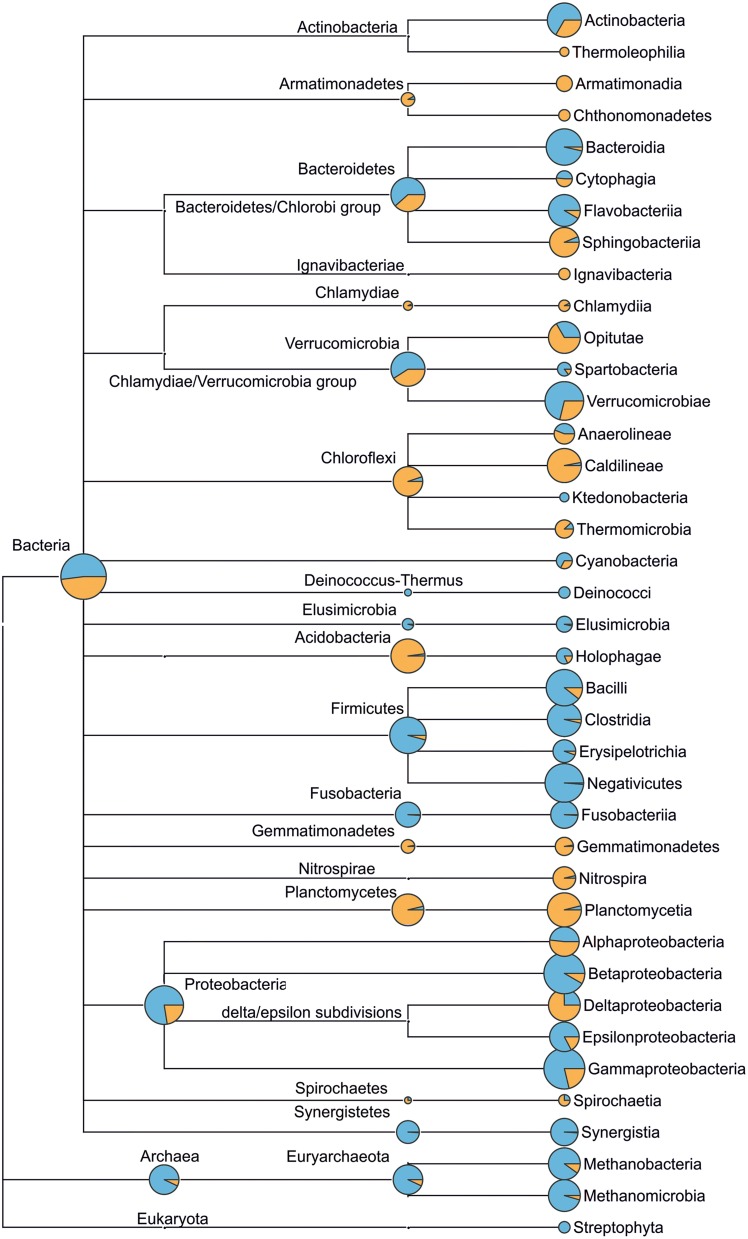
**Microbial community structure (up to *class* taxonomic level) of municipal wastewater (incoming sewage, blue) and WWTP-C (activated sludge, orange) based on 16S rRNA genes sequencing**. The diameter of circles (at class level) is proportional to the logarithm of read count corresponding to the taxon. Circles at higher taxonomic levels (e.g., phylum) reflect the count of reads that cannot be assigned more accurately, namely to lower taxonomic levels (e.g., class). Detailed taxomomic trees (up to genus level) for the incoming sewage and activated sludge of WWTP-C, WWTP-P, WWTP-S are provided in Figures S2–S4.

**Figure 6:**
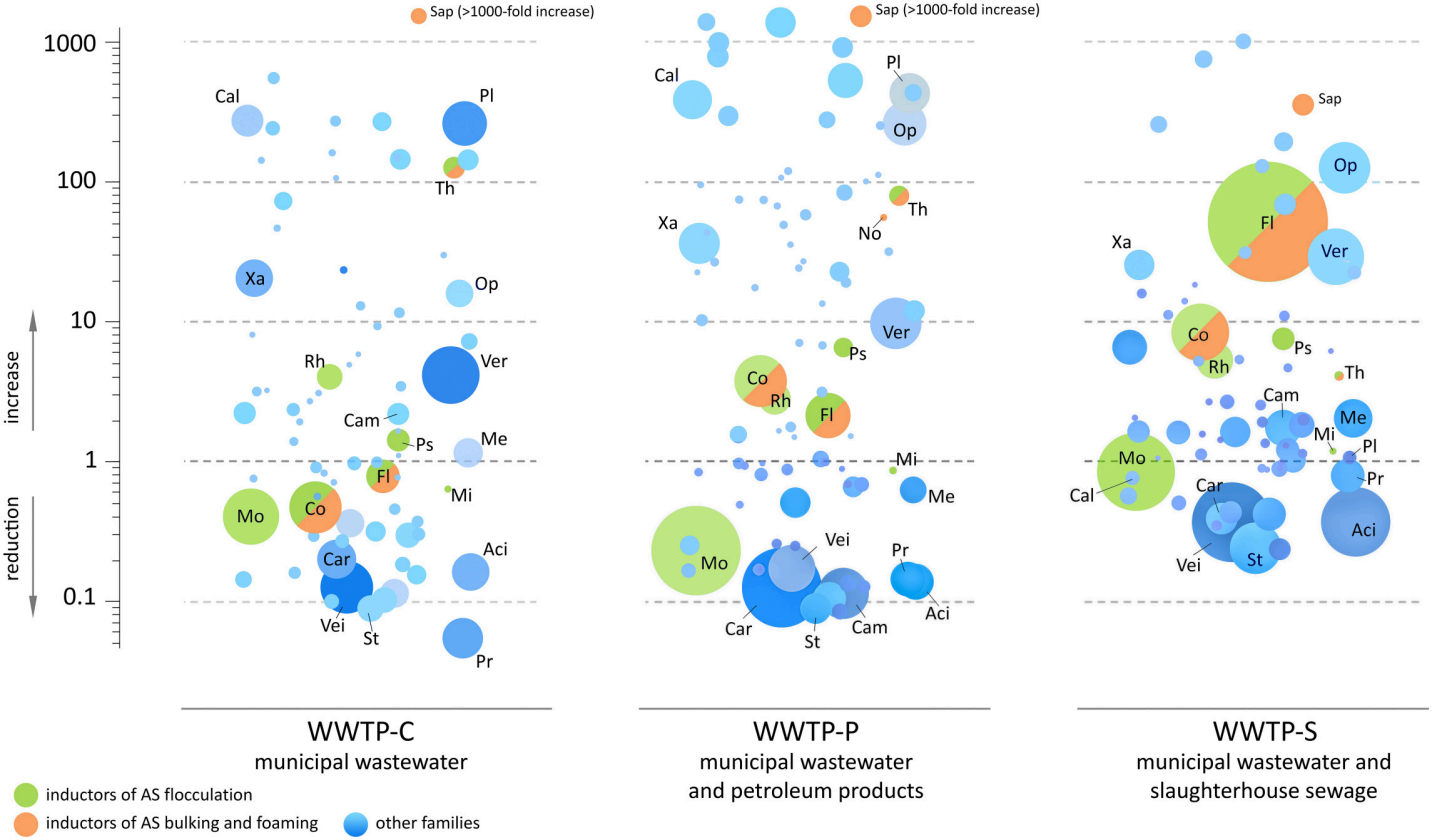
**The estimated changes in the absolute content of bacterial families between the incoming sewage and aerotanks**. Each circle corresponds to one bacterial family. The area of a circle is proportional to the summary read count over the samples of AS and IW for a current WWTP. The vertical position of a circle indicates the estimative change of absolute bacterial content of AS compared to IW: reduction (below zero) or increase (above zero). Designations of taxa: Co, *Comamonadaceae*; Fl, *Flavobacteriaceae*; Mi, *Microbacteriaceae*; Mo, *Moraxellaceae*; No, *Nocardiaceae*; Ps, *Pseudomonadaceae*; Rh, *Rhodocyclacea*; Sap, *Saprospiraceae*; Th, *Thiotrichacea*; Aci, *Acidaminococcaceae*; Cal, *Caldilineaceae*; Cam, *Campylobacteraceae*; Car, *Carnobacteriaceae*; Me, *Methanosarcinaceae*; Op, *Opitutaceae*; Pl, *Planctomycetaceae*; Pr, *Prevotellaceae*; St, *Streptococcaceae*; Vei, *Veillonellaceae*; Ver, *Verrucomicrobiaceae*; Xa, *Xanthomonadaceae*.

The loss of the dominating role in aerotanks was typical of most bacterial species with the highest relative content in the incoming sewage. This is illustrated in Figure 5—the taxonomic tree of BC of municipal wastewater and WWTP-C-AS (detailed trees are presented in Figures S3–S5). However, it would be premature to draw any conclusions concerning a decrease in the absolute number of these bacteria in AS based only on 16S rRNA genes read counts data. The CFU/mL value for an aerotank is 5–12 times higher than that of the incoming sewage (Shchegolkova and Pertseva, 2013). Therefore, before drawing any conclusions concerning the changes in the content of some bacteria in the AS, we should take into account these CFU differences: 16S rRNA genes sequencing only gives a relative taxon content (e.g., proportion), not its absolute value. During the 3-month observation interval (Oct–Dec), WWTP-C sewage contained 1.18 ± 0.23 10^6^ CFU/mL, WWTP-P contained 1.35 ± 0.19 10^6^ CFU/ml, and WWTP-S contained 1.93 ± 0.78 10^6^CFU/mL. AS in the bioreactors contained 11.5 ± 1.89, 14.7 ± 3.34, and 9.64 ± 2.73 CFU/mL, accordingly. We used this data to assess the absolute content of bacterial taxa in the AS and IW samples (Figure S6). However, the derived values are only estimative: they may not represent the absolute bacterial count due to the abundance of non-cultivated species. Moreover, their share may differ between various samples. After performing this “normalization” procedure, we found that the absolute content of the most of abundant bacterial families did not decrease so dramatically in the aerotanks, (up to 10 times; Figure 6, Figure S6), but did change in their proportion (10–100 times; Figures 4, 5).

Only a limited number of bacterial families demonstrated a dramatic decrease in their absolute content: *Succinivibrionaceae, Pasteurellaceae, Carnobacteriaceae*, and others. Some of these families contain known pathogens (including conditional): *Streptococcus, Neisseriaceae* (2–20-fold reduction)*, Leptotrichiaceae* (2–10-fold reduction), *Escherichia*, and *Enterococcaceae* (almost complete elimination). However, the estimated absolute numbers of other families and genera containing pathogenic species were similar or even increased in AS: *Helicobacteraceae, Clostridiaceae, Pseudomonas*, and *Staphylococcaceae*. Generally, the bioreactor of WWTP-S was less effective in disinfection than the other two: *Neisseriaceae, Streptococcus*, and *Leptotrichiaceae* were reduced in quantity only 2- to 3-fold in this aerotank, whereas WWTP-P and WWTP-C reactors demonstrated a 10 to 20-fold reduction in these pathogens.

As can be seen from Figure 6, a significant number of abundant bacterial families did not undergo a dramatic decrease in their absolute numbers in the AS comparing to the IW. A significant part of them represents inductors of AS flocculation (*Moraxellaceae, Comamonadaceae, Flavobacteriaceae, Rhodocyclacea, Pseudomonadaceae, Thiotrichaceacae*), bulking and foaming (*Comamonadaceae, Flavobacteriaceae, Thiotrichaceacae, Nocardioidaceae, Saprospiraceae*). Additional load of bioreactor with petroleum products was associated with 2–4-fold increase in the absolute content of abundant families—*Comamonadaceae, Flavobacteriaceae*, and additional load of bioreactor with organic matter-rich influent—with 8–50-fold increase of the content of these bacteria. However, their proportions did not undergo so dramatic changes (heatmap in Figure 4B). Minor families, *Thiotrichaceacae* and *Saprospiraceae* (inductors of AS bulking and foaming) demonstrated up to 1000-fold increase in their absolute content.

The archaeal community was less diverse than the BC (Figure 5). In all AS samples, we observed a predominance of bacteria over archaea. This was not surprising, as similar results have been published in other studies (Fredriksson et al., 2012; Yu and Zhang, 2012; Wells et al., 2014). The presence of *Thermoprotei, Methanobacteria*, and *Methanomicrobia* classes was found in all three ASs. On the other hand, *Thermoprotei* archaea were detected only in sewage enriched with petroleum products (WWTP-P). The AS and IW of WWTP-C featured the greatest variety of archaea (seven families), whereas two other locations demonstrated lower archaeal biodiversity (only five families).

Detailed data on the taxonomic diversity of the AS and IW BCs are summarized in Table S1. The total number of identified microbial families was similar between all three IW and AS samples of WWTP-C (approx. 160 families). AS of both WWTP-P and WWTP-S demonstrated lower biodiversity at family level: only 145–150 taxa. If we consider the effluent from the slaughterhouse and the refinery to the municipal WWTP as an additional load factor, the simplification of the BC structure (a reduction in the number of families of both bacteria and archaea) in these AS can be explained by this factor. On this basis, it can be argued that the number of identified taxa (families, genera) is a diagnostic marker of the BC condition. A reduced diversity of BCs may indicate the appearance of an additional undesirable load factor. However, this question requires further investigation; the impact of various loads on microbial community of laboratory-scale WWTPs needs to be evaluated. Under the environmental conditions, the reduction of biodiversity in aquatic communities is often associated with an increase in the load on any factor, for example the emergence of toxicants, xenobiotics, or organic matter, (Sobczyñski and Joniak, 2008).

The sequences are available at NCBI Sequence Read Archive (study SRP067575, BioProject PRJNA306487).

### Analysis of the metabolic potential of activated sludge bacterial communities

**Figure 7:**
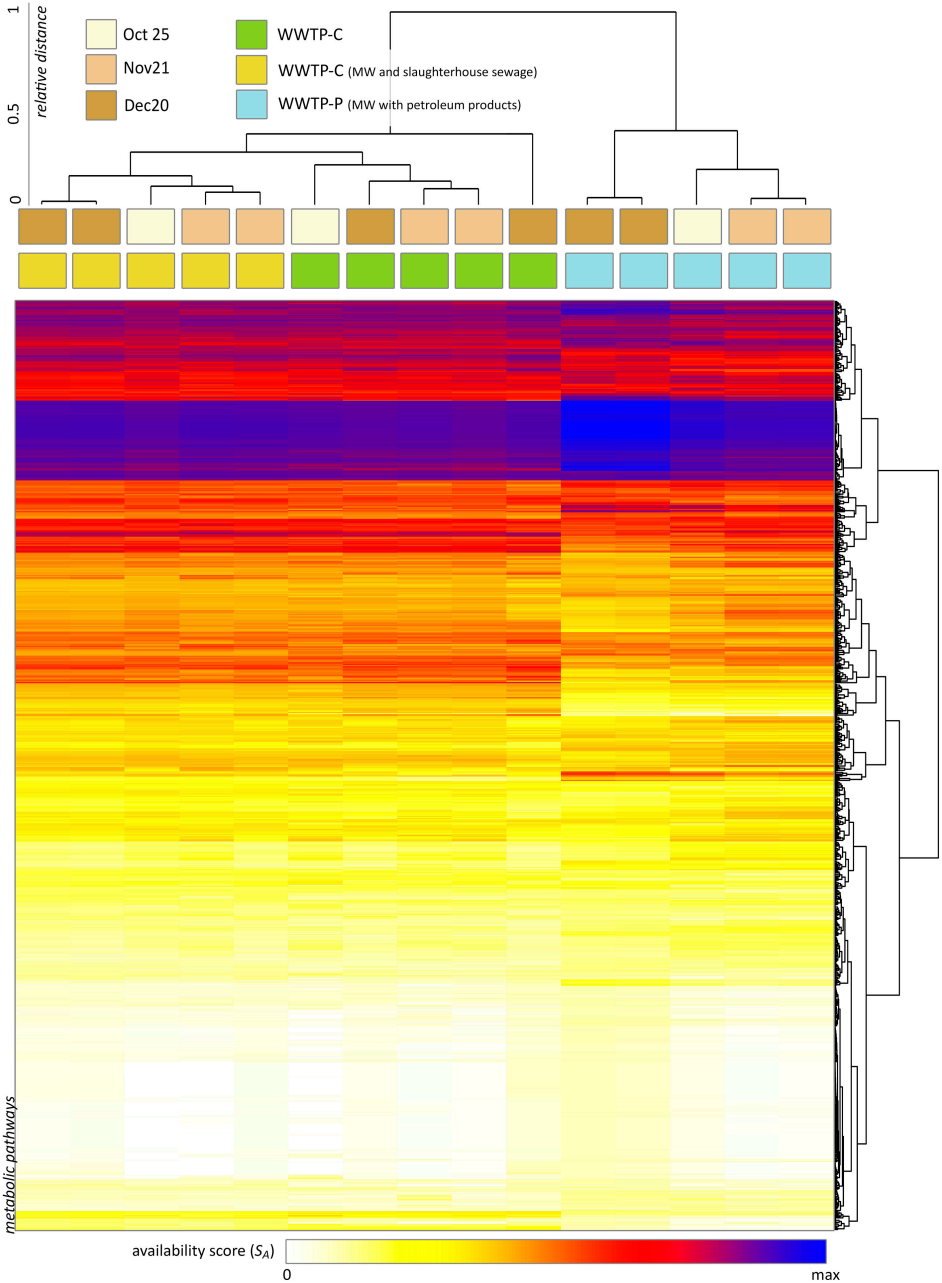
**The results of metabolic capacity analysis across 15 samples of AS (performed with XeDetect)**. Heatmap indicates the square root of the availability score *S*_*A*_ reflecting the representativity of 1500 MetaCyc pathways in the current bacterial community. Cluster analysis of the samples suggests the similarity between almost all specimens related to the same WWTP.

Using XeDetect, we evaluated the *availability score* for MetaCyc pathways across 15 samples of AS. About 1500 pathways and superpathways were found to be completely available in these communities (*S*_*A*_ > 0, Figure 7). The most striking differences noted were:

- AS of WWTP-S (performing treatment of slaughterhouse wastewater) shows the greatest difference from the other two WWTPs.
- There was an increased *S*_*A*_ for amino acid degradation pathways in WWTP-S.
- *S*_*A*_ for the glyoxylate cycle and fatty acid degradation superpathway in WWTP-S is four to five times greater than that for other WWTPs. This effect is mostly due to the increase in *Flavobacteriaceae*.
- We observed a preference for different metabolic pathways between different WWTPs—for example, leucine degradation III pathway (to methylbutanol) had a 3–10 times greater *S*_*A*_ in WWTP-C and WWTP-P compared to that in WWTP-S. However, the leucine degradation I pathway (to acetoacetate) had tw*S*_*A*_ in WWTP-S-AS twice as large as that in WWTP-C and WWTP-P.
- The processes of ammonification and nitrification were slightly elevated in WWTP-S and WWTP-P.

Complete data are provided in Table S2.

## Discussion

AS has been known as a microbial community, self-assembled according to the environmental conditions of the bioreactor, long before the era of high-throughput metagenomic studies (Seviour and Nielsen, 2010). In practice, until recently, conclusions drawn from the dynamics of AS structure and its sustainability have been based only on the evaluation of input–output balance of pollutants, direct counts of bacteria, and sowing on selective media (MacConkey agar, mannitol salt agar, xylose lysine desoxycholate, etc.). The introduction of novel high-throughput sequencing approaches has enabled the rapid and effective taxonomic profiling of AS microorganism communities, and has made it possible to evaluate their temporal dynamics (Lee et al., 2015).

Wells et al. (2014) studied the transfer of microorganisms between distinct units of WWTP—trickling filter biofilm, trickling filter effluent, AS, and plant influent (Wells et al., 2014). The authors concluded that this process could have a significant effect on microbial-community dynamics within staged bioreactors. The authors demonstrated that microbial immigration between coupled process units has potentially profound implications for bioprocess engineering and control (Wells et al., 2014). However, the quantitative assessment of this effect is still to be made. A year later, Lee et al. analyzed the biodiversity of IW and AS from the WWTPs that treat mostly domestic wastewater (Lee et al., 2015). In the AS, they found a loss or decline in most bacterial taxa that are abundant in the incoming sewage. However, 5–10% of OTUs remained at similar levels in the AS.

In the present work, we revealed the relative stability of AS BCs over time for three full-scale WWTPs performing detoxification of wastewater with different compositions and origins, i.e., conventional municipal sewage; municipal wastewater with refinery sewage, rich in petroleum products; and slaughterhouse wastewater, containing increased amounts of bioorganic matter. For each type of wastewater, *Acinetobacter* (*Moraxellaceae* family) was found to be the most abundant bacterial genus. This is in agreement with the findings of other authors (Vandewalle et al., 2012). *Acinetobacter* is a strictly aerobic chemoorganotrophic bacterium with an oxidative metabolism. We found several wastewater type-specific minor bacterial taxa (e.g., the presence of Rhodocyclaceae in the WWTP that treat MW, and MW with petroleum products), which can serve as a unique bacterial signature of each wastewater type. Long-term stability of AS taxonomic profiles was significantly greater than that of IWs (*p* = 0.01), and the dissimilarity of AS to different WWTPs was more pronounced. In contrast to the incoming sewage, each bioreactor was characterized by its own set of most abundant bacterial families. This taxonomic signature did not significantly vary over time (Figure 4A). To support this conclusion, we performed principal component analysis of the taxonomic compositions of AS and IW samples (Figure S7). As can be seen from Figure S7, AS samples comprise three groups on the plot (the 2^nd^ and 3^rd^ principal components). Two of these groups, WWTP-C and WWTP-P are located close to each other, whereas AS from WWTP-S shows the greatest distance from the other two. This signature was stable over time. Our results are in accord with the previous findings of other authors—the microbial composition of the AS was found to be unique for each plant and constant over time, giving a characteristic plant-specific “fingerprint” (Mielczarek et al., 2012).

The principles of AS microbial community formation should serve as a basis for the efficient monitoring and regulation of AS structure. This allows extending AS biodegradational capability, and avoiding many technical problems. The elimination of various pollutants, toxicants, and xenobiotics is a growing problem due to the increase in drugs and other chemicals available in the market (Devillers et al., 2013). The biodegradation of most xenobiotics requires a cascade of reactions. Such cascade is only possible in a community of microorganisms with complex trophic relations.

Various approaches, experimental techniques, prediction models, and databases enabling the evaluation of xenobiotic biodegradability have been suggested (Devillers et al., 2013; Hoffmann et al., 2014). The prediction of the biodegradational capability of AS or other BCs is directly related to the analysis of their metabolic networks. However, the only method currently available for comprehensive analysis of BC metabolic potential is complete metagenome sequencing, followed by the evaluation of the abundance of enzyme-coding genes, and the reconstruction of the available pathways (Inskeep et al., 2013). However, metagenome sequencing is an expensive procedure, so it cannot be performed for regular monitoring of AS biological structures.

XeDetect bridges the gap between the MetaCyc database (Altman et al., 2013; Caspi et al., 2014) and the taxonomic composition of a BC. This allows a set of available enzyme-catalyzed reactions to be derived and, in turn, determines a set of metabolic pathways responsible for the degradation of various compounds. Based on the differences in the chemical composition of the IW described above, we should expect the abundance of bacteria responsible for the biodegradation of fatty acids, proteins, and other biological material in WWTP-S AS to be greater than that in the other two WWTPs. Indeed, XeDetect analysis revealed increased availability scores for the pathways of amino acids and fatty acid degradation in WWTP-S, and elevated *S*_*A*_ for toxicant degradation pathways (phenols, toluene, etc.) in WWTP-P (the complete list of availability scores is presented in Table S2).

MetaCyc is an extensive database, though not covering all the enzyme-catalyzed reactions in each microorganism. However, XeDetect-mediated MetaCyc analysis allows us to reveal the differences in metabolic capabilities between distinct BCs, and to highlight the toxicants that could be biodegraded in their current microbial environments. Certainly, there are some limitations. The major limitation arises from ignoring the *u*_*i, j*_ coefficients in the formula for *v*. Hence, it is preferable to compare the availability scores *S*_*A*_ for the same pathway in distinct microbial communities, rather than to compare *S*_*A*_ in two or more distinct metabolic pathways. In addition, we should take into account the fact that, although *S*_*A*_ reflects the availability of the metabolic pathway, the pathway could be inactive due to the lack of substrate. However, these two limitations are also present in the methods of inferring pathways based on complete metagenome sequencing. Another major limitation arises from the impossibility of exact taxonomic annotation of BCs at species or strain level. Different strains of one bacterial species can dramatically differ in their biodegradational capacity for various compounds (Itoh et al., 2013; Tomas-Gallardo et al., 2014). Moreover, the horizontal gene transfer may have its effect.

As mentioned above, most bacterial families that are abundant in IW have significantly decreased in their relative content in the AS. For example, we observed decrease in the absolute content of Acinetobacter (*Moraxellaceae*), which plays a significant role in the detoxification of different pollutants, such as degradation of aromatic compounds (Mazzoli et al., 2007; Felföldi et al., 2010). However, some major bacterial taxa either increased or remained in the aerotanks at stable levels. These are *Comamonadaceae, Pseudomonadaceae, Verrucomicrobiaceae*, and *Flavobacteriaceae*. These families (along with *Moraxellaceae*) are the major components of AS of the most WWTP worldwide. They play crucial roles in degradation of organic compounds and forming floc structure of AS. Moreover, many inductors of AS bulking and foaming efficiently degrade stable organic compounds and their increase in content and biodiversity is reasonable (Caravelli et al., 2007; Jin et al., 2011; Phuong et al., 2012; Yadav et al., 2012). In itself, their abundance in AS suggests their involvement in the trophic chains of BC and, therefore, indicates the presence of degradable substrates in the incoming sewage. Indeed, XeDetect analysis suggests that an increase in *Flavobacteriaceae* is a major cause of the elevated *S*_*A*_ of fatty acid, lipid, and protein degradation pathways in WWTP-S. The excessive abundance of such bacteria is associated with AS bulking and foaming (Jin et al., 2011; Guo et al., 2015), but their complete elimination or significant decrease would mean a loss of AS biodegradation capability. Therefore, one of the pivotal questions related to WWTP operation is that pertaining to the optimization of the balance between the degradational potential, and an excessive number of bacteria responsible for the serious technical problems in AS operation. XeDetect analysis would help to evaluate this balance, and suggest metabolic pathways responsible for the degradation of key pollutants based on the taxonomic composition of AS.

One of the tasks of a WWTP is the disinfection of wastewater. Ultraviolet radiation and chemical agents are most commonly used for these purposes (Aslani et al., 2014; Childress et al., 2014). Both these procedures are expensive, and the latter, chemical wastewater treatment, may be harmful for the environment. Recent studies have raised the opportunity of so-called biological disinfection—the elimination of pathogenic microorganisms in active biological environments, including the AS of WWTP (Ivanov et al., 2010). We observed a dramatic reduction of *Streptococcus, Neisseriaceae, Leptotrichiaceae, Escherichia*, and *Enterococcaceae* in the AS of aerotanks. However, WWTPs with incoming slaughterhouse wastewater showed the least effective disinfection: our analysis revealed that there was only a twofold decrease in the abundance of most of the mentioned families (in absolute counts). XeDetect analysis suggested the involvement of these microorganisms in the degradation of bioorganic compounds, abundantly represented in WWTP-S sewage (Table S3). Thus, the excessive load of biological material in WWTPs may cause some technical problems such as AS bulking and foaming. Moreover, it impairs elimination of pathogenic bacteria.

## Conclusions

The chemical, rather than the biological composition of IW, is a major factor in the formation of AS taxonomic structure. AS demonstrated stable over time taxonomic pattern, whereas the incoming sewage did not. The specific taxonomic structures were revealed in AS, which are influenced by wastewater rich in bioorganic matter (slaughterhouse wastewater) and oil products (refinery sewage). In addition, we found that most of the pathogenic microflora (*Streptococcus, Neisseriaceae, Leptotrichiaceae, Escherichia*, and *Enterococcaceae*) were significantly diminished in the aerotanks.

We have developed XeDetect tool to enable the analysis of BC metabolic capacity. In contrast to other tools, the XeDetect approach only requires BC taxonomic structural data. XeDetect analysis suggested a high availability score of metabolic pathways responsible for the degradation of fatty acids and amino acids, and other biological material in the reactor of WWTP-S (treating municipal wastewater and slaughterhouse wastewater), and an elevated score for pathways of the degradation of toxicants (phenols, toluene, etc.) in WWTP-P (treating municipal wastewater and refinery sewage). The WWTP-S reactor demonstrated an increase in bacteria responsible for AS bulking and foaming, two major problems in WWTP operation. XeDetect analysis found these organisms to play an important role in the degradation of the bioorganic substrates that are a part of incoming sewage. The excessive quantity of biological material (fatty acids, lipids, and proteins) in incoming sewage may create conditions for the growth of inductors of AS bulking and foaming and adversely affects the diversity of the AS BC and the disinfection capability of the bioreactor.

Regular monitoring of AS taxonomic structure and evaluation of metabolic potential with XeDetect analysis could help optimize bioreactor performance, and avoid some of the most common technical problems.

## Author contributions

NS, GK, and AK conceived and designed the work. AB, SK, KK, NM, and AK performed the experiments. NS, GK, and AD analyzed the data. NS, GK, and AD drafted the work. All authors revised the work critically for important intellectual content, approved the version to be published, and agreed to be accountable for all aspects of the work in ensuring that questions related to the accuracy or integrity of any part of the work are appropriately investigated and resolved.

## Funding

This work was performed under the financial support by the Russian Science Foundation (Grant 14-17-00672).

### Conflict of interest statement

The authors declare that the research was conducted in the absence of any commercial or financial relationships that could be construed as a potential conflict of interest.

## Supplementary material

Figure S1 **Screenshot of XeDetect graphical user interface**. The right panel illustrates the taxonomic structure of the bacterial community being analyzed, and the left panel represents a list of available metabolic pathways. The availability score *S*_*A*_ is represented in S_a column.

Figure S2 **Distance matrices for the activated sludge (AS) and incoming wastewater samples (IW)**. All taxonomy levels are included in the distance calculation. Replicates (the samples taken with 15 min interval) are shown separately (left panel) and are averaged (right panel). Boxes indicate samples taken from one spot. The average distances for each group of samples are shown. One can see a twofold increase in the average distance for the IW samples comparing to AS. This indicates greater time-stability of AS samples compared with IW (*p* = 0.01).

Figures S3–S5 **Taxonomic trees illustrating the differences between the structures of incoming wastewater (light brown) and activated sludge (lilac) bacterial communities of three WWTPs**. The diameter of circles is proportional to the logarithm of reads count for these taxa including all subsidiaries (normalized by library size).

Figure S6 **The taxomic structure of activated sludge and incoming wastewater (data are re-normalized taking into account CFU/ml differences between the samples). (A)** Dendrogram illustrating the results of hierachial clustering analysis of AS and IW samples. **(B)** Heatmap reflecting the abundance of microorganisms. Families containing pathogens, inductors of AS flocculation, bulking and foaming are marked with colored squares.

Figure S7 **Principal component analysis of taxomonic structure of incoming wastewater and activated sludge samples**. A triplet of squares represents one probe. IN, incoming wastewater; AS, activated sludge; WWTP-C, city—municipal wastewater; WWTP-S, sla—MW and slaughterhouse sewage; WWTP-P, petro—MW, and refinery sewage.

Table S1 **The taxonomic composition of AS and IW: read counts for taxa**.

Table S2 **Metabolic potential of AS of three WTTPs: normalized availability scores of metabolic pathways annotated in MetaCyc, and separately—pathways responsible for the degradation of various compounds**.

Table S3 **Metabolic pathways of pathogenic microorganisms found in the activated sludge**. Columns: the name of a pathway; the availability of a key reaction; S_a—availability score (*S*_*A*_); ID—MetaCyc identifier. Yellow color indicates incomplete pathways. Green-blue gradient reflects the value of *S*_*A*_.
