# Supplementary figures and images for: Microbial Community Structure of Activated Sludge in Treatment Plants with Different Wastewater Compositions
Source: Front Microbiol. 2016 Feb 18;7:90. doi: 10.3389/fmicb.2016.00090 (PMC4757684; doi:10.3389/fmicb.2016.00090)

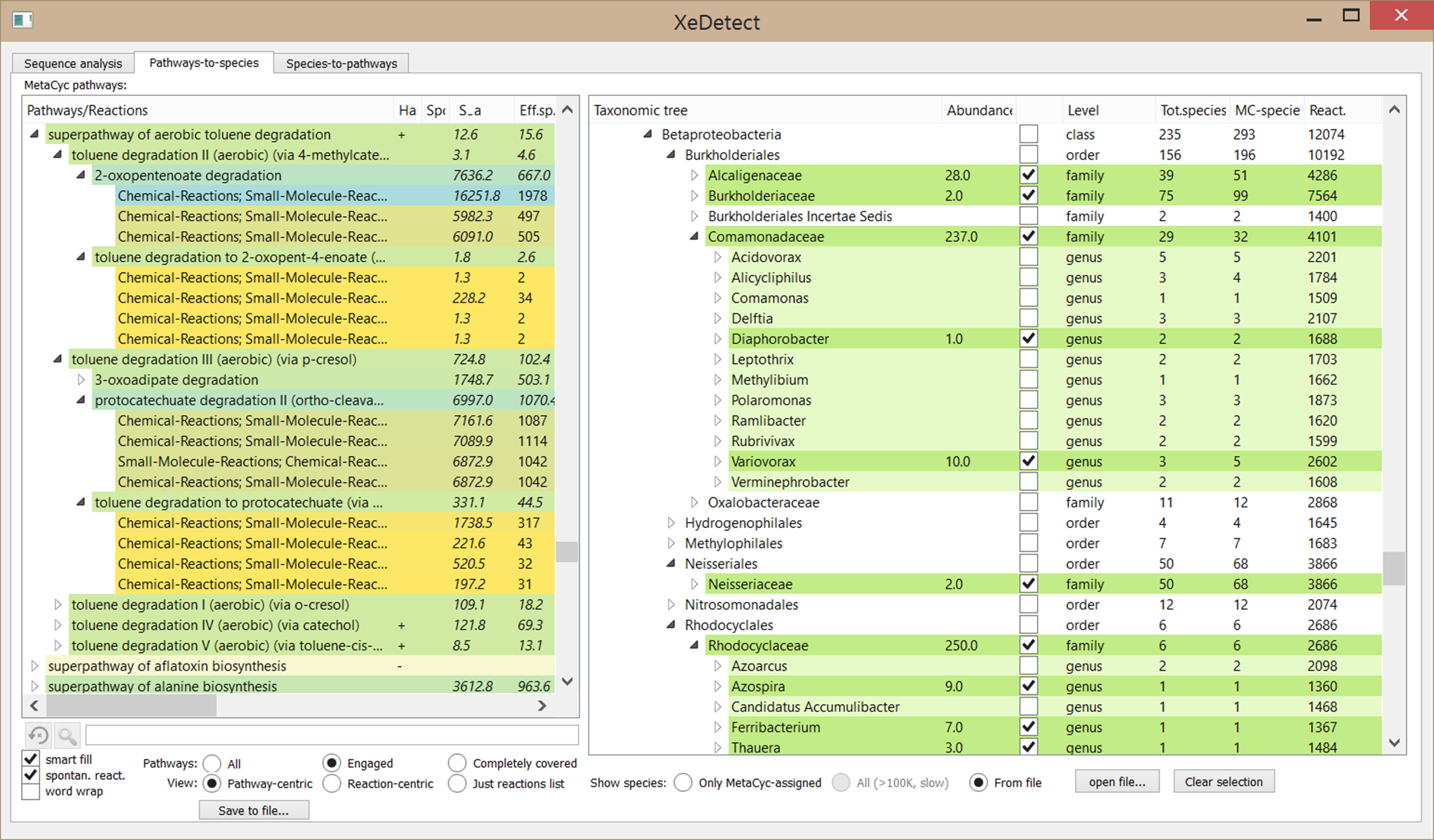

Supplement: Figure S1 — Screenshot of XeDetect graphical user interface. The right panel illustrates the taxonomic structure of the bacterial community being analyzed, and the left panel represents a list of available metabolic pathways. The availability score SA is represented in S_a column. [file Image1.TIF]

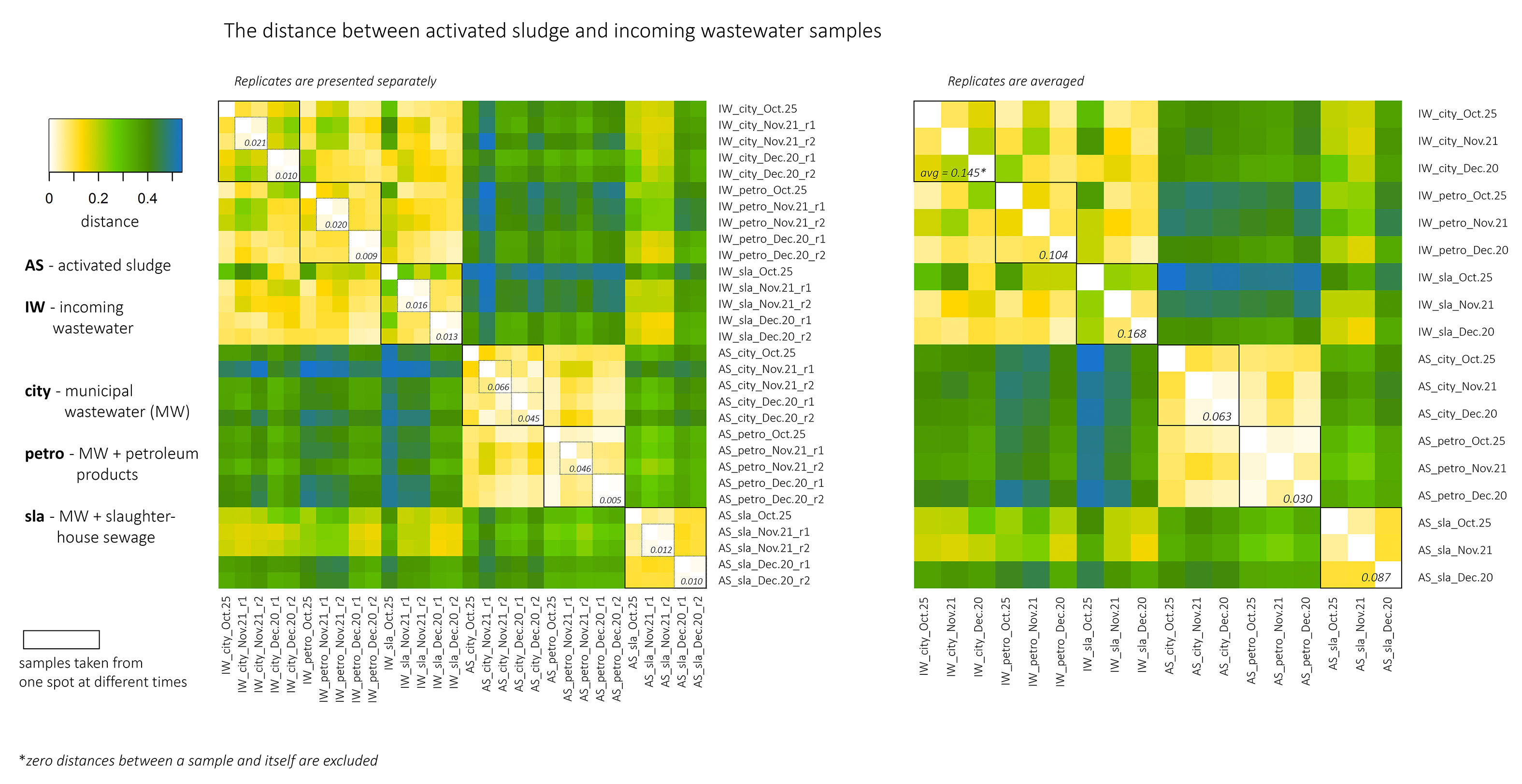

Supplement: Figure S2 — Distance matrices for the activated sludge (AS) and incoming wastewater samples (IW). All taxonomy levels are included in the distance calculation. Replicates (the samples taken with 15 min interval) are shown separately (left panel) and are averaged (right panel). Boxes indicate samples taken from one spot. The average distances for each group of samples are shown. One can see a twofold increase in the average distance for the IW samples comparing to AS. This indicates greater time-stability of AS samples compared with IW (p = 0.01). [file Image2.TIF]

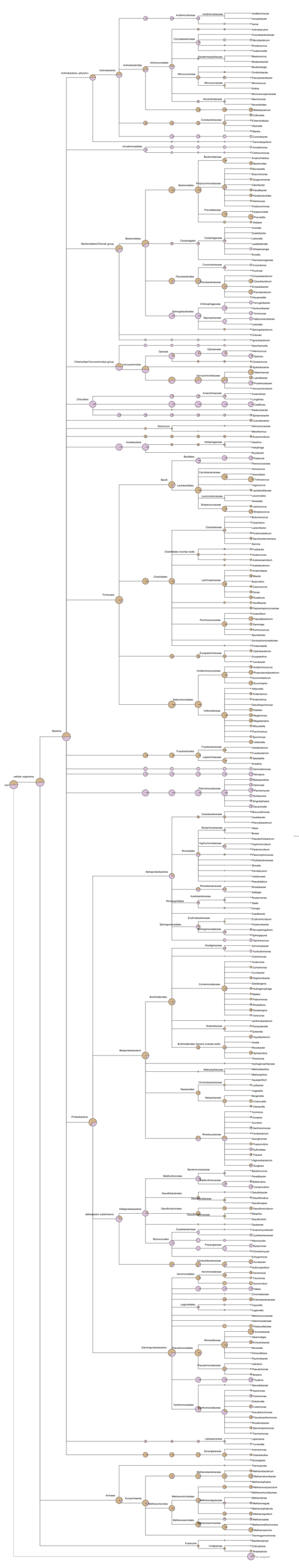

Supplement: Supplementary file 3 [file Image3.PDF]

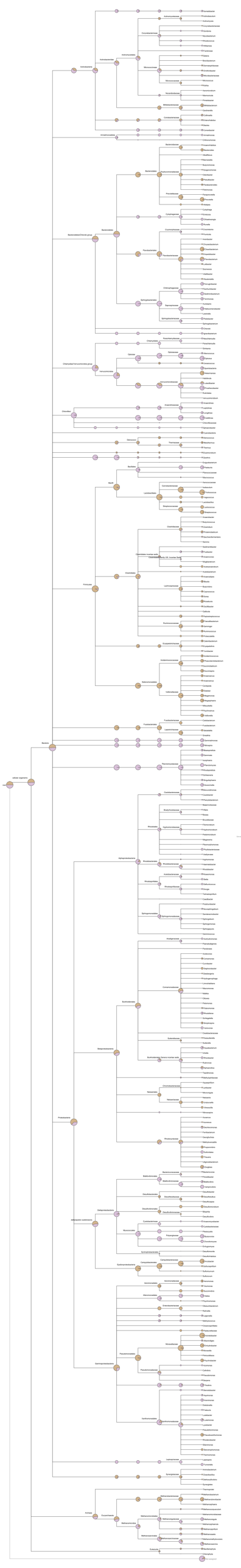

Supplement: Supplementary file 4 [file Image4.PDF]

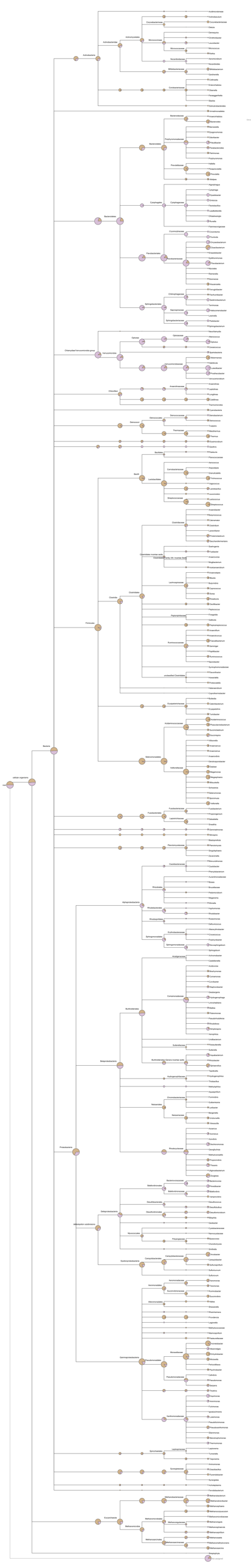

Supplement: Figures S3–S5 — Taxonomic trees illustrating the differences between the structures of incoming wastewater (light brown) and activated sludge (lilac) bacterial communities of three WWTPs. The diameter of circles is proportional to the logarithm of reads count for these taxa including all subsidiaries (normalized by library size). [file Image5.PDF]

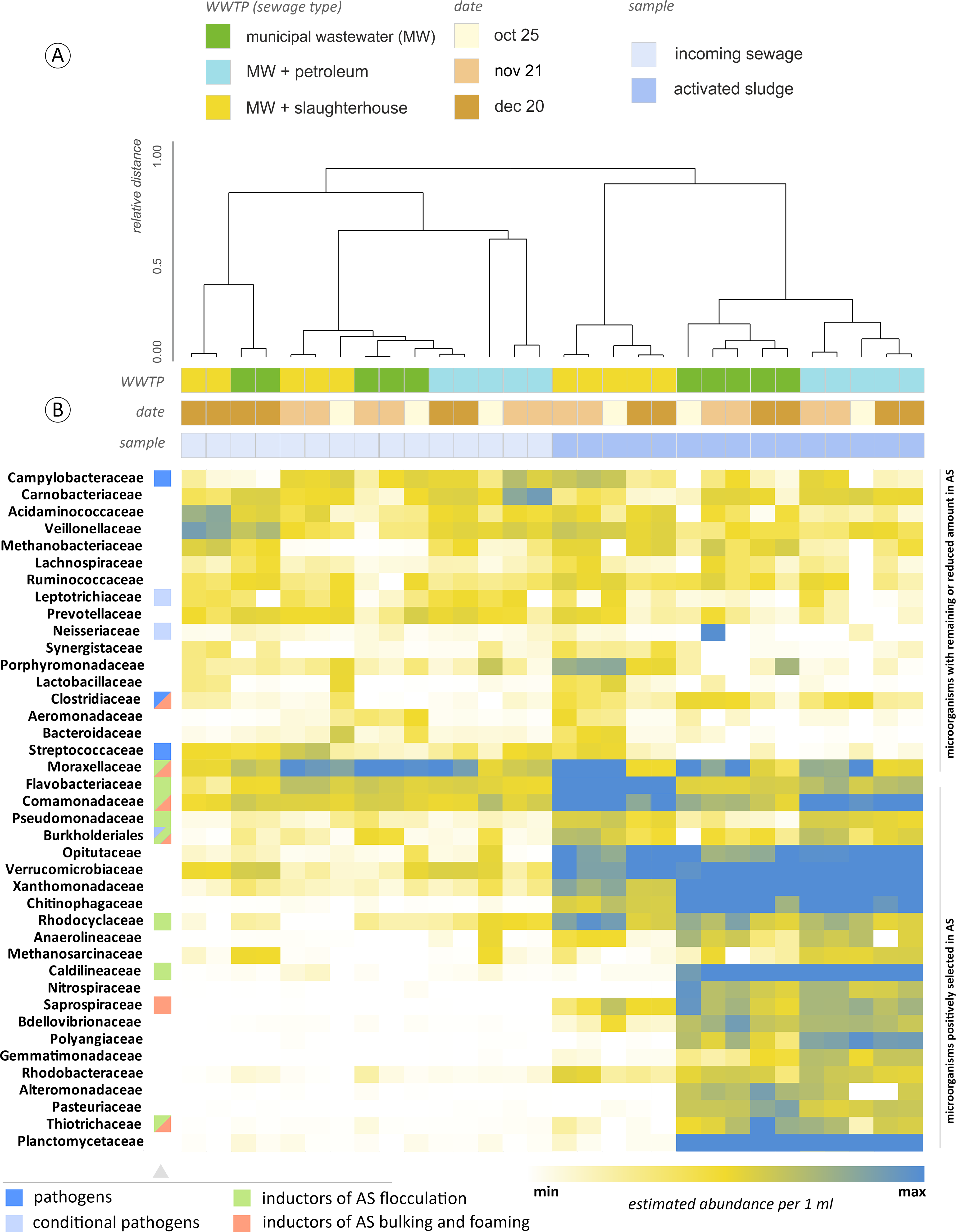

Supplement: Figure S6 — The taxomic structure of activated sludge and incoming wastewater (data are re-normalized taking into account CFU/ml differences between the samples). (A) Dendrogram illustrating the results of hierachial clustering analysis of AS and IW samples. (B) Heatmap reflecting the abundance of microorganisms. Families containing pathogens, inductors of AS flocculation, bulking and foaming are marked with colored squares. [file Image6.tif]

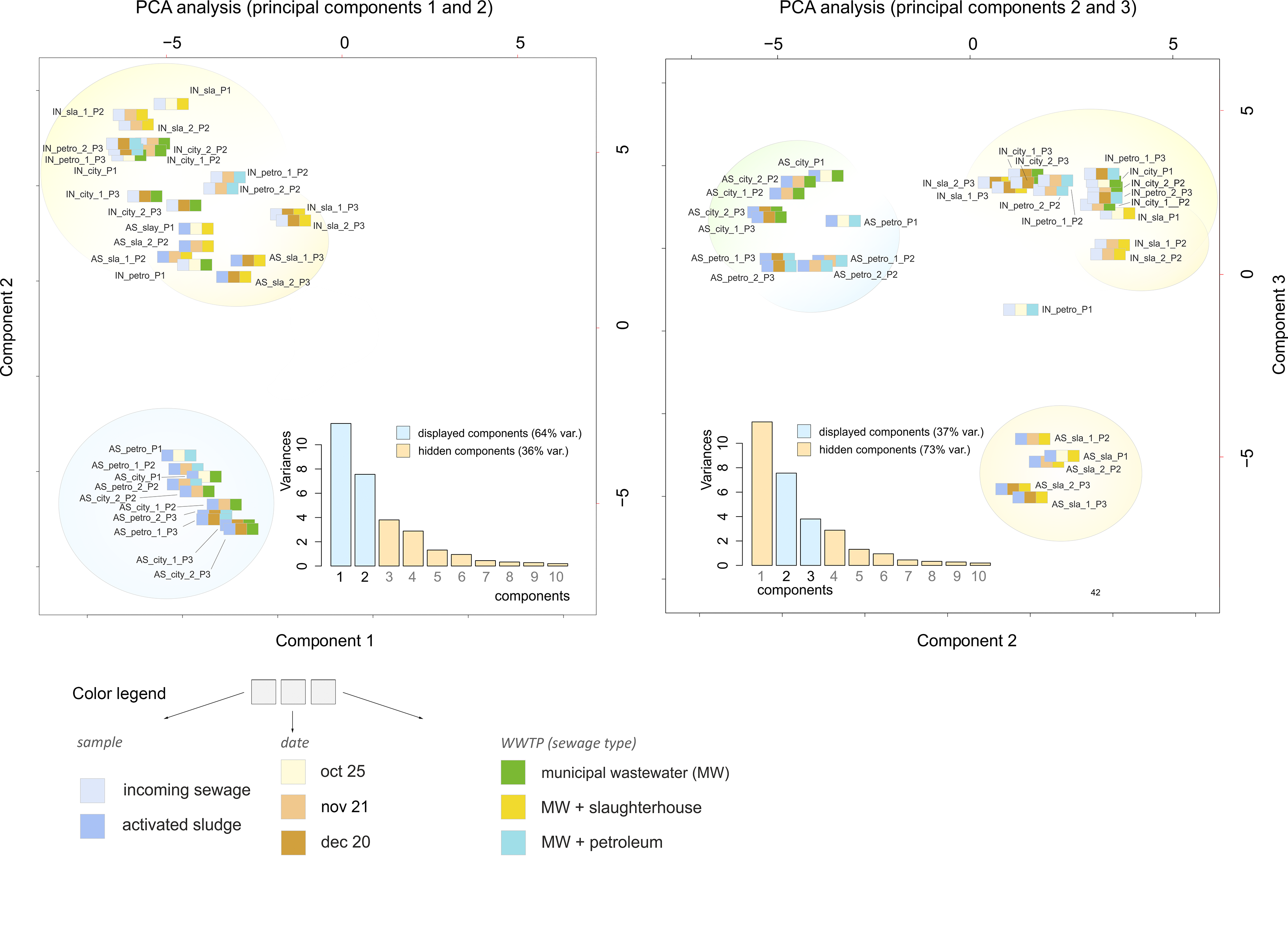

Supplement: Figure S7 — Principal component analysis of taxomonic structure of incoming wastewater and activated sludge samples. A triplet of squares represents one probe. IN, incoming wastewater; AS, activated sludge; WWTP-C, city—municipal wastewater; WWTP-S, sla—MW and slaughterhouse sewage; WWTP-P, petro—MW, and refinery sewage. [file Image7.TIF]
